# Effects of different irrigation protocols on dentin surfaces as revealed through quantitative 3D surface texture analysis

**DOI:** 10.1038/s41598-020-79003-9

**Published:** 2020-12-16

**Authors:** Shlomo Elbahary, Sohad Haj-yahya, Majd Khawalid, Igor Tsesis, Eyal Rosen, Waseem Habashi, Ariel Pokhojaev, Rachel Sarig

**Affiliations:** 1 Department of Endodontics, The Maurice and Gabriela Goldschleger School of Dental Medicine, Sackler Faculty of Medicine, Tel Aviv University, 69978 Tel Aviv, Israel; 2 The Maxillofacial Surgery and Dental Clinic Department, Shamir Medical Center, 70300 Zrifin, Israel; 3 Tel Aviv University Center for Nanoscience and Nanotechnology, 69978 Tel Aviv, Israel; 4 Department of Oral Biology, the Maurice and Gabriela Goldschleger School of Dental Medicine, Sackler Faculty of Medicine, Tel Aviv University, 69978 Tel Aviv, Israel; 5 The Dan David Center for Human Evolution and Biohistory Research, Sackler Faculty of Medicine, Tel-Aviv University, 6997801 Tel Aviv, Israel

**Keywords:** Root canal treatment, Endodontics, Confocal microscopy

## Abstract

The combination of ethylenediaminetetraacetic acid (EDTA) and sodium hypochlorite (NaOCl) has been advocated as an effective irrigation methodology to remove organic and inorganic matter in root canal therapy. Yet, it was suggested that both solutions might lead to structural changes of the dentinal wall surface, depending on the order of application which might affect sealer mechanical retention. This study aims to evaluate the effect of different irrigating protocols on dentin surface roughness using quantitative 3D surface texture analysis. Data stems from 150 human root dentin sections, divided into five groups, each prepared according to one of the following protocols: Negative control; 17% EDTA; 17% EDTA followed by 5.25% NaOCl; 5.25% NaOCl; and 5.25% NaOCl followed by 17% EDTA. Each dentin sample was examined for its three-dimensional surface texture using a high-resolution confocal disc-scanning measuring system. EDTA 17% and the combined EDTA 17% with NaOCl 5.25% showed considerably higher roughness properties compared to the control and to NaOCl 5.25% alone. However, the irrigation sequence did not affect the dentin roughness properties. Therefore, mechanical retention is probably not dependent upon the selection of irrigation protocol sequence.

## Introduction

One of the main challenges in endodontic treatment is to achieve complete sealing^1^ of the root-canal, yet, currently used materials and obturation techniques are often not sufficient. Indeed, microleakage remains a clinical problem and a possible source of failure^2^. The most common method for root-canal obturation includes the use of a core material in combination with a root-canal sealer^1^.

Adequate sealing ability and sufficient adhesive strength are required properties for sealer efficacy^1,3^. Root canal sealer adhesive strength depends on chemical and mechanical retention. A smooth surface is more efficient in case of chemical bonding, providing better adhesion. Yet, micromechanical bonding requires the presence of surface irregularities into which the adhesive can penetrate^1,3^. These irregularities can be quantified by measuring surface roughness, which is a component of surface texture that is evaluated by the deviation of an ideal surface from the real surface. Specifically, if these deviations are large, the surface is rough; if they are small, the surface is smooth^4^.

Researchers suggested that irrigation in root canal therapy can lead to structural changes of the dentin walls of the root and changes in surface roughness^5–8^. Therefore, consideration should be given to appropriate selection of dentine preparation and irrigation protocols to match the sealer type and to allow adequate mechanical and chemical retention^1,3^.

An integral part of root canal treatment is the use of irrigations to disinfect the root canal system and to remove debris and tissue remnants^9,10^. The current methods of root canal preparation might produce a smear layer that covers the instrumented areas of the canal walls^11^. The smear layer contains inorganic and organic substances such as fragments of odontoblastic processes and necrotic debris^9^, and may also contain bacteria. It may act as a physical barrier and affect the sealing efficiency of the root canal filling. To avoid these consequences, irrigation is used to remove the smear layer^10,12^, thus requiring the use of a chelating agent and a soft-tissue solvent^13^. The combination of ethylenediaminetetraacetic acid (EDTA) and sodium hypochlorite (NaOCl) was suggested as an effective irrigation procedure to disinfect the root canal and to eliminate the organic and inorganic materials^9^. However, the ideal irrigation sequence, volume, and application time remain disputed in the literature^14–16^. In most cases, NaOCl is used during instrumentation, and EDTA is preferably used at the end of instrumentation to complete the removal of the smear layer^14,15^. The application of NaOCl ensures a high disinfection efficacy and enables the material to penetrate into the dentin. In contrast, a final flush of NaOCl has also been suggested to allow better NaOCl penetration to areas that were earlier covered with the smear layer^16^.

Structural and surface changes in the dentin may contribute to the development of root fractures; hence, they might bear significant clinical implications^17–19^. On the other hand, it was reported that applying EDTA and NaOCl solutions to the root canal resulted in an eroded appearance of the dentin and enlarged tubular orifice diameters^20,21^. This could be of clinical significance as in the case of the alteration micromechanical bonding of the adhesive materials. Therefore, the aim of the current study was to evaluate the effect of irrigation protocols on dentine surface roughness.

Scanning electron microscopy (SEM) is one of the most commonly used methods for the qualitative study of dentin erosion^17,22–26^. However, it lacks the ability to image the third dimension, which allows evaluation of material loss after applying different irrigation protocols. Quantitative methods commonly used to describe dentin roughness include 2D contact profilometry (i.e., the surface is scanned with a stylus equipped with a diamond or steel tip)^27^ or atomic force microscopy (AFM)^22^. However, contact profilometry allows 2D profile analysis (rather than surface) and AFM allows evaluation of only relatively small surface areas^22^. The arithmetic average roughness parameter (Ra), which is the most frequently reported profile roughness measurement in dental literature, may thus provide incomplete or insufficient information about the nature of area surface characteristics.

New technologies based on scale-sensitive fractal analysis of high-resolution and three-dimensional surface reconstructions have advanced the ability to produce accurate, measurable analysis for surface texture^28,29^. Similarly, quantitative 3D surface texture analysis (3DST) has become an established method for dietary reconstruction by analyzing dental surfaces^30,31^. Surface texture analysis was used to analyze tooth surfaces for various purposes in evolutionary biology studies and has opened up opportunities for various applications in clinical practice^28,32^. 3DST analysis utilizes standardized parameters from engineering applications (ISO 25178-2^33^), allowing reliable characterization of tooth attrition in a micrometer scale. 3DST analysis of the wear and the change in surface texture can reveal the mechanical process^28,32,34^ and the physical properties of the contacting materials^35^.

Indeed, 3DST, a non-contact profilometry, allows better flexibility for analyzing very deep erosion pits and even curved natural surfaces in 3D manner^27^. Therefore, the battery of parameters used in the present study provides more comprehensive analysis of the surface pattern including height as well as spatial, hybrid, function and segmentation features: Parameters belonging to the Height subgroup solely depend on the statistical distribution of z-axis values of the measured surface. Periodic motifs in the lateral directionality of the data, if present, are described by spatial parameters. Hybrid parameters rely on a combination of texture amplitude and spacing measurements to enable differentiation of surfaces exhibiting similar average roughness. Functional (volume) parameters indicate a measure of material void volume, based on the Abbott-Firestone areal material ratio curve. Feature subgroup employ the watershed algorithm to identify and characterize the prominent surface features (dales and hills).

Here, we aimed to evaluate the effect of different irrigating protocols on dentin surface texture by assessing the dentin roughness using quantitative 3DST.

## Materials and methods

### Preparation of dentin specimens

The study was approved by the Tel Aviv University Ethics Committee (approval number 230.17), and all methods were performed in accordance with the relevant guidelines and regulations. Informed consent was obtained from all participants included in the study.

**Figure 1:**
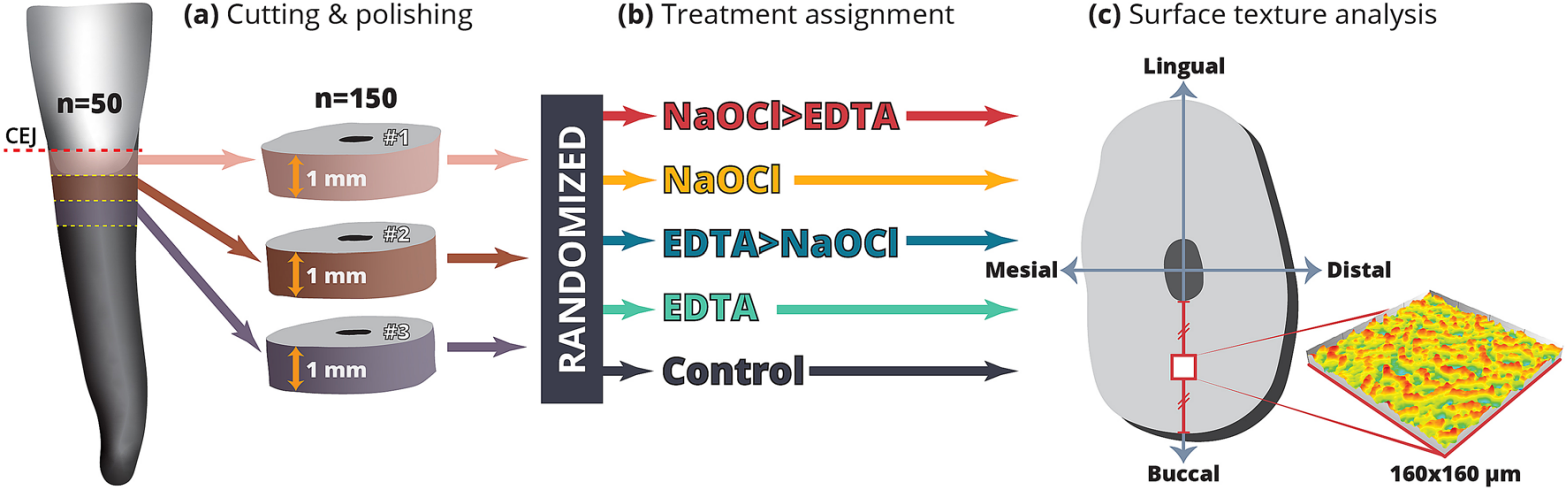
Scheme of the experimental protocol. (**a**) For each tooth (n = 50), three slices, each 1 mm thick, were cut using a low-speed diamond saw (Isomet, Buehler Ltd., Lake Bluff, IL, USA) and ground to achieve a smooth surface using 400-, 600-, 800- and 1200-grit polishing papers. (**b**) A total of 150 dentin slices were randomly assigned to 5 treatment groups (containing 30 slices each). (**c**) For each slice, the midpoint between the pulp chamber and the buccal-most point was identified along the bucco-lingual axis to obtain a 160 × 160 µm surface measurement using a high-resolution confocal disc-scanning measuring system (100 × long distance lens, μsurf explorer, NanoFocus AG, Germany). Subsequently, quantitative 3D surface texture analysis per spot of 160 × 160 µm was conducted using the Mountains Map Premium software (v 7.3.7, DigitalSurf, France).

Fifty fully-developed human permanent anterior teeth were used in this study. All teeth were freshly extracted for periodontal reasons from male and female patients (age range 30–45 years). Teeth with previous root canal treatment, root resorption, or caries, as well as teeth with root fractures, were excluded from the study. Once the tooth was extracted, the debris and soft tissue remnants were removed from the root with a sharp scalpel. Then the teeth were stored in phosphate-buffered saline until used for the study. For preparation of the specimens, the crowns were removed by a cut at the cemento-enamel junction using a high-speed tungsten 28 mm Zekrya bur (De-Trey, Dentsply, Konstanz, Germany) under water coolant. The remaining roots were sectioned perpendicular to the long axis of the root under water cooling with a diamond saw rotating at 500 rpm (Isomet, Buehler Ltd., Lake Bluff, IL, USA). Each root was cut to produce three slices 1 mm thick each (150 dentin slices in total, Fig. 1a). To achieve a smooth surface, the slices were ground using 400-, 600-, 800- and 1200-grit polishing papers consecutively under distilled water, allowing to remove any surface irregularities. After each grinding step, dentin samples were rinsed and ultra-sonicated in purified water.

The 150 dentin slices were randomly divided into five equal groups, with slices obtained from the same tooth separated into different groups. Each group was subjected to one of the following irrigation protocols (Fig. 1b): (1) Negative control—saline solution (sodium chloride 0.9%) (Baxter. Inc. Deerfield, Il, USA) for 10 min; (2) (E) 17% EDTA (Sigma-Aldrich, Inc., St. Louis, MO, USA) for 10 min; (3) (EN) 17% EDTA for 10 min followed by 5.25% NaOCl (Sigma-Aldrich, Inc.) for 10 min; (4) (N) 5.25% NaOCl for 10 min; (5) (NE) 5.25% NaOCl for 10 min followed by 17% EDTA for 10 min. All specimens were immersed in 1.8 ml volume of solution and were shaken for 20 s every minute. The protocol was based on previous studies showing that cleansing by NaOCl and EDTA and removal of the smear layer were most efficient at an application time of 10 min^36–38^. After removal from the irrigation solution, the samples were extensively rinsed and kept in deionized and distilled water for no longer than 20 min prior to further analysis.

### Surface texture analysis

Surface texture analysis of the dentin slices was conducted under 1000 × magnification using a high-resolution confocal disc-scanning measuring system (μsurf Explorer, NanoFocus AG, Germany) equipped with a 160 L lens (100 ×, long distance). The configuration of the system yields a lateral resolution of 310 nm and a height resolution of < 3 nm for the 160 × 160 μm measured area.

For each slice, the midpoint between the pulp chamber and the buccal-most point was identified along the bucco-lingual axis (Fig. 1c), and a quantitative 3D surface texture analysis per a spot of 160 × 160 µm was conducted^39^. Surface roughness was measured using the Mountains Map Premium software (v 7.3.7, DigitalSurf, France). The parameters used for evaluation of the surface roughness were based on standardized ISO parameters (ISO 25178-2) (Table 1), each representing distinctive characteristics of the surface texture^40^.

**Table 1:** Descriptive statistics (mean, standard deviation = SD) are given for surface texture parameters (ISO 25178-2) measured of the five groups (1—CONTROL, 2—EDTA, 3—EDTA + NaOCl, 4—NaOCl, 5—NaOCl + EDTA). *p*-value for zone-wise comparisons resulting from the ANOVA are shown, level of significance was set to *p* < 0.05 and Post Hoc (Tukey) analysis show the significant differences between the three areas.

Surface texture parameter	Units	CONTROL (1)		EDTA (2)		EDTA + NaOCl (3)		NaOCl (4)		NaOCl + EDTA(5)		Without control		With control	
		Mean	SD	Mean	SD	Mean	SD	Mean	SD	Mean	SD	Sig	Post hoc	Sig	Post hoc
**Height**															
Root-mean-square height (*Sq*)	µm	1.01	0.29	0.97	0.18	1.08	0.25	1.01	0.30	1.30	0.35	0.001	2, 4, 3 ≠ 5	0.003	2, 4, 1 ≠ 5
Skewness (*Ssk*^a^)		0.18	0.75	− 0.19	0.17	− 0.19	0.25	0.08	0.49	− 0.14	0.19	NS		NS	
Kurtosis (*Sku*^a^)		4.33	1.31	3.27	0.29	3.31	0.34	3.65	1.32	2.93	0.43	0.05	5 ≠ 4	0.001	5, 2, 3 ≠ 1
Maximum peak height (*Sp*^a^)	µm	6.88	1.97	4.62	1.08	5.43	1.74	5.97	2.14	5.32	1.41	0.017	2 ≠ 4	< 0.001	2, 5 ≠ 1
Maximum pit height (*Sv*)	µm	4.57	1.40	5.00	0.69	5.52	0.83	4.15	1.19	5.44	0.84	< 0.001	4 ≠ 2, 5, 3	< 0.001	4 ≠ 5 1 ≠ 3
Maximum height (*Sz*)	µm	11.45	2.55	9.69	1.70	11.49	2.73	10.15	2.61	10.66	2.02	NS		NS	
Arithmetic mean height (*Sa*)	µm	0.79	0.24	0.77	0.14	0.86	0.21	0.81	0.25	1.04	0.27	0.002	2, 4, 3 ≠ 5	0.003	2, 1, 4 ≠ 5
Areal material ratio (*Smr*)	%	0.01	0.01	0.02	0.01	0.01	0.01	0.01	0.01	0.03	0.03	0.049	3, 4 ≠ 5	0.024	1, 4 ≠ 5
Inverse areal material ratio (*Smc*)	µm	1.24	0.40	1.20	0.22	1.34	0.30	1.27	0.40	1.65	0.52	0.002	4, 2, 3 ≠ 5	0.004	2, 1, 4 ≠ 5
Extreme peak height (*Sxp*)	µm	1.98	0.76	2.05	0.44	2.10	0.40	1.86	0.57	2.46	0.45	0.002	4, 2, 3 ≠ 5	0.014	4, 1 ≠ 5
**Spatial**															
Autocorrelation length (*Sal*^a^)	µm	63.83	30.23	33.58	29.59	33.56	30.73	63.37	38.19	49.77	38.38	0.002	3, 2 ≠ 4	0.001	3, 2 ≠ 4, 1
Texture-aspect ratio (*Str*)		0.47	0.22	0.36	0.21	0.38	0.21	0.42	0.26	0.37	0.25	NS		NS	
Texture direction (*Std*^a^)		86.41	22.57	85.65	42.88	62.41	28.30	98.37	20.98	94.34	8.57	0.001	3 ≠ 2, 5, 4	0.002	3 ≠ 4, 5
**Hybrid**															
Root-mean-square gradient (*Sdq*^a^)		0.42	0.07	0.63	0.07	0.63	0.08	0.42	0.07	0.58	0.07	< 0.001	4 ≠ 5, 2, 3 5 ≠ 3	< 0.001	1, 4 ≠ 5, 2, 3
Developed interfacial area ratio (*Sdr*)	%	7.93	2.41	16.59	3.36	16.79	3.62	8.05	2.37	14.37	3.06	< 0.001	4 ≠ 5, 2, 3 5 ≠ 2, 3	< 0.001	1, 4 ≠ 5, 2, 3
**Functional (volume)**															
Material volume (*Vm*)	µm^3^/µm^2^	0.05	0.02	0.04	0.01	0.04	0.01	0.04	0.01	0.04	0.01	NS		NS	
Void volume (*Vv*)	µm^3^/µm^2^	1.29	0.41	1.24	0.23	1.38	0.31	1.31	0.41	1.61	0.43	0.016	2, 4 ≠ 5	0.037	2, 1 ≠ 5
Peak material volume (*Vmp*)	µm^3^/µm^2^	0.05	0.02	0.04	0.01	0.04	0.01	0.04	0.01	0.04	0.01	NS		NS	
Core material volume (*Vmc*)	µm^3^/µm^2^	0.87	0.28	0.87	0.16	1.02	0.30	0.89	0.27	1.17	0.29	< 0.001	4, 2 ≠ 5	0.002	2, 1, 4 ≠ 5
Core void volume (*Vvc*)	µm^3^/µm^2^	1.17	0.39	1.12	0.21	1.29	0.32	1.20	0.39	1.45	0.42	0.024	2 ≠ 5	NS	
Pit void volume (*Vvv*)	µm^3^/µm^2^	0.12	0.05	0.12	0.03	0.12	0.02	0.11	0.04	0.14	0.02	0.011	4 ≠ 5	NS	
**Feature**															
Density of peaks (*Spd*)	1/µm^2^	0.01	0.00	0.01	0.00	0.01	0.00	0.01	0.00	0.01	0.00	< 0.001	4 ≠ 5, 3, 2 5, 3 ≠ 2	< 0.001	1, 4 ≠ 5, 3 2 ≠ 1, 4, 5
Arithmetic mean peak curvature (*Spc*)	1/µm	1.35	0.23	1.66	0.25	1.83	0.27	1.41	0.25	1.73	0.29	< 0.001	4 ≠ 2, 5, 3	< 0.001	1, 4 ≠ 2, 5, 3
Ten point height (*S10z*)	µm	7.28	1.67	6.51	0.78	7.85	1.15	5.57	1.39	6.46	1.36	< 0.001	4 ≠ 5, 3, 2 5, 2 ≠ 3	< 0.001	1 ≠ 4 3 ≠ 2, 5, 3
Five point peak height (*S5p*)	µm	4.24	1.22	3.02	0.44	3.65	0.79	2.88	0.93	3.00	0.41	0.004	4, 5, 2 ≠ 3	< 0.001	4 ≠ 3 1 ≠ 2, 5, 4
Five point pit height (*S5v*)	µm	3.04	1.00	3.42	0.46	4.20	0.71	2.73	0.93	3.50	0.74	< 0.001	4 ≠ 2, 5, 3 2, 5 ≠ 3	< 0.001	4 ≠ 2, 5 3 ≠ 1, 2, 4, 5
Mean dale area (*Sda*)	µm^2^	193.85	60.59	72.26	17.44	102.24	34.40	148.21	56.88	93.70	19.45	< 0.001	3, 5 ≠ 4 2 ≠ 3, 4	< 0.001	2, 5, 3 ≠ 4 1 ≠ 2, 3, 4, 5
Mean hill area (*Sha*^a^)	µm^2^	189.76	57.95	77.41	17.79	102.26	37.04	136.65	49.41	92.68	23.02	< 0.001	2, 3, 5 ≠ 4	< 0.001	2, 5 ≠ 4 1 ≠ 2, 3, 4, 5
Mean dale volume (*Sdv*)	µm^3^	15.59	7.86	5.83	1.24	10.47	4.60	9.42	4.83	7.54	2.45	< 0.001	2 ≠ 4, 3 5 ≠ 3	< 0.001	2 ≠ 3 1 ≠ 2, 3, 4, 5
Mean hill volume (*Shv*)	µm^3^	13.87	5.92	6.70	1.67	8.82	3.70	8.56	3.96	7.37	2.29	NS		< 0.001	1 ≠ 2, 3, 4, 5

^a^Larger value shows less roughness.

Statistical analysis was carried out using SPSS (v. 21.0). Significance was set to p < 0.05. Outliers were removed from the analysis using the ROUT method (Q = 1%)^41^. Kolmogorov–Smirnov tests were carried out to verify the normality of the measurement distributions. For parametric measurements (with normal distribution), one-way analysis of variance (ANOVA) with post-hoc (Tukey) tests was carried out. For non-normally distributed parameters, Kruskal–Wallis analysis with post-hoc (Bonferroni) tests was used. Between-group principal component analysis (PCA) of the measurements that significantly differed between the studied zones (p < 0.05, Table 1) was carried out using the PAST software (v.3.16^42^).

### Scanning electron microscopy (SEM) analysis

One cross-section of radicular dentin from each treatment group was retrieved for SEM analysis. The sections were mounted on an aluminum stub using a conductive adhesive and coated with gold–palladium using the sputtering machine. The sections were then imaged using SEM (JOEL JSM-IT100 PLUS) to obtain high-resolution images in secondary electrons (SE) mode using 20 kV. All images were obtained at the midpoint between the pulp chamber and the buccal-most point along the bucco-lingual axis. The images were performed under 1000 × and 2000 × magnification.

## Results

The mean and standard deviation of roughness values of dentin surfaces for the five treatment groups are listed in Table 1. The experimental groups (i.e., E, N, EN, NE) showed significantly (p < 0.001) higher roughness than the control group in most measured parameters (Table 1). This higher roughness was particularly apparent in the features parameters (i.e., S5p, Sda, Sha, Sdv, Shv; see Table 1 for list of abbreviations, p < 0.001). Other parameters (e.g., Smr, Sxp, Sal, Sdq, Sdr, Spd, Spc) showed some similarity between the control group and the N group.

**Figure 2:**
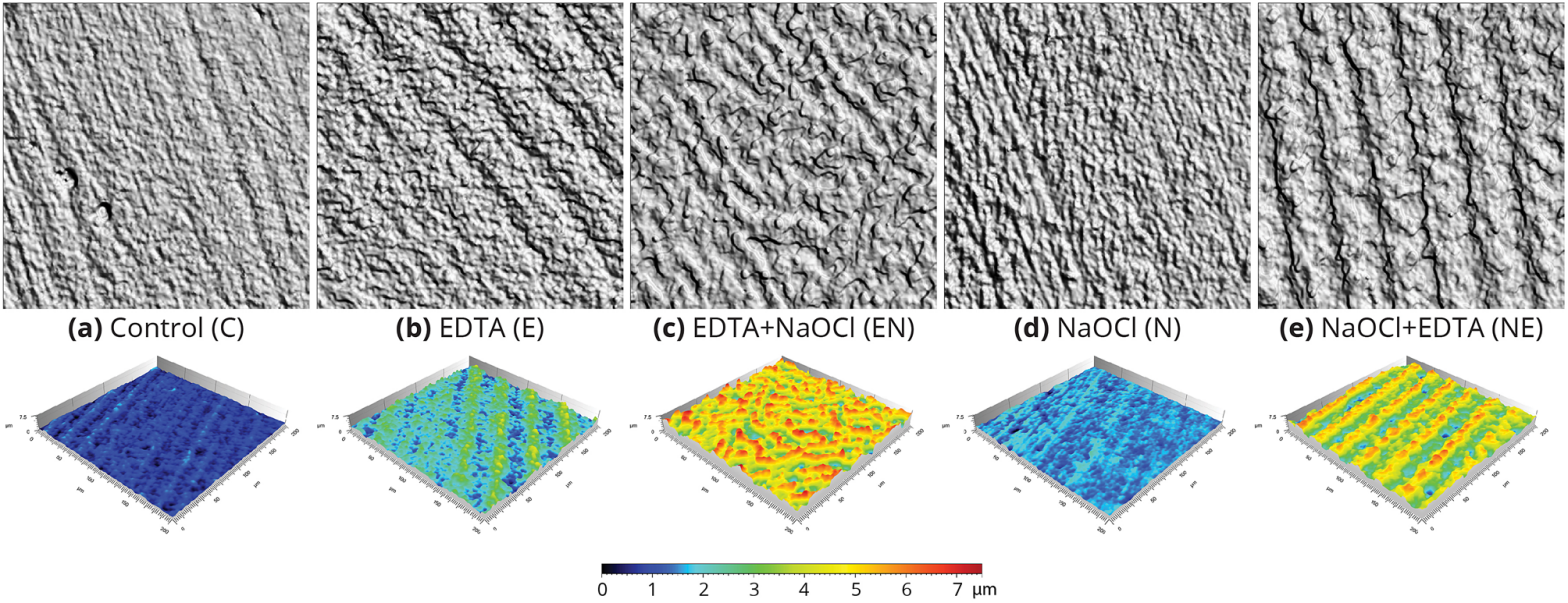
Surface image (top) and surface topography (bottom) of the dentin surfaces following the irrigation protocols. A 160 × 160 μm image was acquired using a high-resolution confocal disc-scanning measuring system (100 × long distance lens, μsurf Explorer; NanoFocus AG). (**a**) Control; (**b**) EDTA 17%; (**c**) EDTA 17% followed by NaOCl 5.25%; (**d**) NaOCl 5.25%; (**e**) NaOCl 5.25% followed by EDTA 17%. Note the reduced roughness of the control (**a**) and NaOCl 5.25% (**d**) groups compared to the other treatments (**b**, **c**, **e**). The color map indicates the measured height in micrometers above the lowest point for each surface.

**Figure 3:**
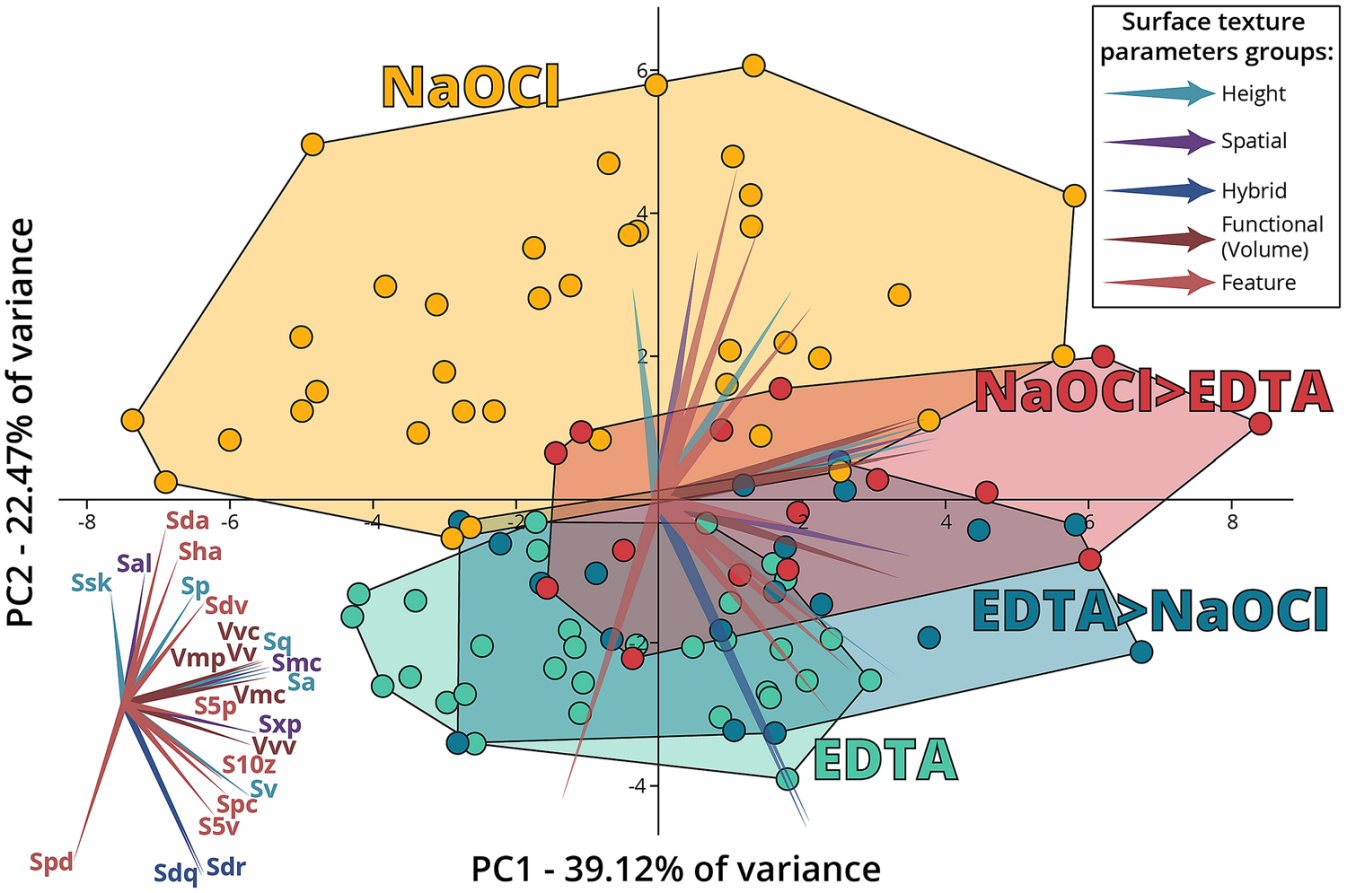
Between-group PCA of the dentin surface roughness parameters. Graphic presentation of the overall difference between the four experimental groups (E, EN, N and NE). The first two principal components explain 61.59% of the variance of the measured surface texture parameters (ISO 25178-2^24^). The list of abbreviations is listed in Table 1. Each parameter is designated as a vector that indicates the contribution of the parameter to the difference between the regions. The parameters are divided into five groups (each highlighted by a different color) according to the surface pattern (see Table 1).

The protocols of EDTA 17% and EDTA 17% combined with NaOCl 5.25% (E, EN, NE) showed significantly higher roughness properties compared to NaOCl 5.25% alone (N) in 10 out of 30 parameters (Sv, Sal, Sda, Sdr, Spd, Spc, S10z, S5v, Sda and Sha) (p < 0.001, Table 1, Figs. 2, 3). The order of the irrigation for the NE and EN groups did not affect most of the surface roughness properties. In other words, the protocol of NaOCl 5.25% following 10 min EDTA 17% treatment did not significantly change the surface texture compared to the protocol of adding EDTA 17% following 10 min of NaOCl 5.25% (p > 0.05). Nineteen out of 30 parameters (Ssk, Sku, Sp, Sy, Sz, Sal, Str, Vm, Vy, Vmp, Vmc. Vvc, Vyy, Spd, Spc, S10z, Sda, Sha and Shy) showed no significant differences in this regard.

To describe the four treatment protocols graphically and to demonstrate the overall effect of the measured parameters, we used principal component analysis (PCA) for the parameters that showed significant differences (p < 0.05, 23 parameters, Table 1). The first two principal components account for 61.59% of the variance of the measured surface texture parameters (ISO 25178-2). The graph represents the differences between the groups according to each parameter directionality (Fig. 3). Based on the graph, the protocol of NaOCl only (N) clearly differs from the other protocols mainly in the parameters along the PC2 axis (which represents 22.47% of the variability of the results). These parameters can mostly account for height and feature parameters (e.g., Ssk, Sal, Sda, Sha, Sp and Sdv). The overlap between the NE and EN protocols (Fig. 3) indicates that most of the parameters did not show any significant difference in surface roughness.

**Figure 4:**
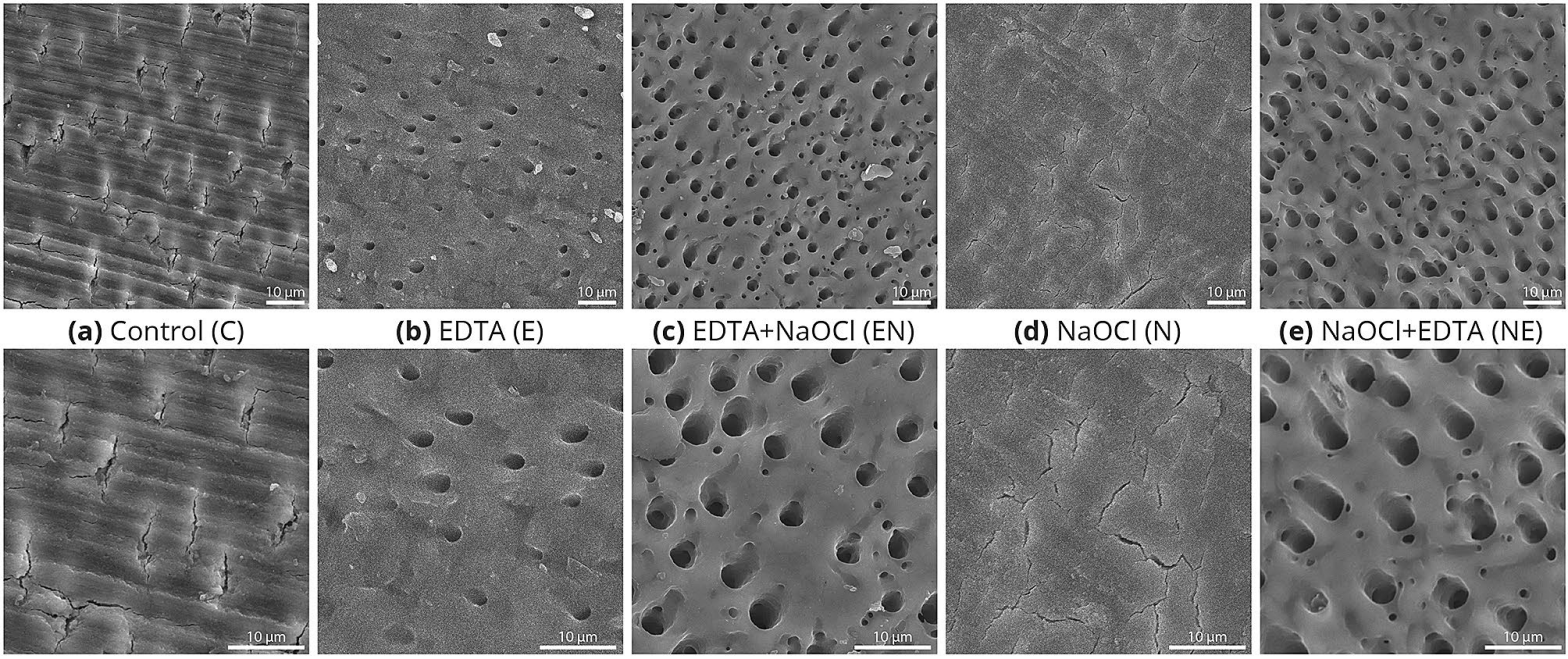
SEM images of the dentin surfaces following the different irrigation protocols. × 1000 (top row) and × 2000 magnifications (bottom row). (**a**) Control; (**b**) EDTA 17%; (**c**) EDTA 17% followed by NaOCl 5.25%; (**d**) NaOCl 5.25%; (**e**) NaOCl 5.25% followed by EDTA 17%. Scale bar: 10 µm.

The SEM images of the control group (C) evidently revealed a cracked smear layer, which completely covered the dentinal tubules (Fig. 4). The N specimens show similar features of surface coverage, with no open tubules. The E group show several tubules opening, with some depositions on the surface, yet, with no apparent smear layer. In the EN group, clearly opened tubules were observed and the smear layer appeared to be completely removed, with slight deposition on the surface. The NE group produced similar results, showing clearly open tubules with no smear or debris. Yet, unlike the EN group, the surface lacked any other depositions.

## Discussion

In the present study, we used a new methodology, 3DST, to evaluate the effect of different irrigation protocols used in root canal treatments on the dentin surfaces by assessing changes in dentin roughness, which can reflect the surface properties and characteristics. Surface roughness is one of the test methods most frequently used to evaluate the effects of different irrigations on dental tissues^5,22,43^.

3DST was previously suggested as a reliable method to characterize wear and changes of surface texture in human enamel^28,32^. Yet, this is the first study to analyze the dentin surface through this prism. The main advantage of the 3DST method is that it allows reliable characterization of tooth surfaces at high resolution, both qualitatively and quantitatively. The data attained can shed light on the mechanical properties and function of the studied surface^34,35^.

Using the 3DST method, we were able to analyze the dentin surface directly, before and after several irrigation procedures. This is the first study to utilize all 30 standardized ISO parameters for dentin roughness evaluation. Thus, the effect of each irrigation solution could be analyzed and the dentin roughness could be described.

The addition of surface metrology to this field of research is of great importance since it allows standardization of measurements and comparison of future studies. Moreover, with the development of new materials and enhancement of current protocols, the standardized surface parameters may be the only objective, replicable manner of testing the efficiency of newly-designed materials compared to the currently-used substances.

The model chosen for the study was the dentin disc model, with an irrigation duration of 10 min. A clinical trial by Byström and Sunvquist^36^ proved that cleansing using NaOCl and EDTA was efficient after 10 min, and that the removal of the smear layer by EDTA and NaOCl was efficient following a 10-min exposure^38^. It was also suggested that the demineralization process of the dentin by EDTA is a time-dependent process, and the critical time was shown to be 10 min^37^.

The dentin disc has been extensively used as a reliable model for assessing and screening potential desensitizing agents in vitro^44,45^. The main advantage of the dentin disc model is that each disc exhibits variations in size, density and orientation of the tubuli. Therefore, this model can represent a broad spectrum of effects on the complex dentinal system composed of canaliculi that branch off from the main tubules at a variety of angles^46^. The mechanical preparation method (i.e., manual or rotatory) is known to increase the root canal area, thus exposing the tubuli from different angles. Applying an irrigation solution following mechanical preparation might result in penetration of the solution in various directions, not necessarily along the dentinal tubuli. That said, one of the limitations of this model is that the dentinal tubules in the blocks may have openings from both ends, which may facilitate rapid penetration of the solutions into the dentin. Obviously, this model does not mimic the exact clinical situation; however, it can assist in revealing the effect of irrigation solutions on dentin.

Dentin is composed of an organic core-dense collagen network, covered by an inorganic outer sheath hydroxyl apatite coating^17^. NaOCl, which is a strong base and nonspecific oxidizer, causes degradation of the amino acids by neutralization and chloramination reactions^25^. Type I collagen and glycosaminoglycan have been found to lose their immunoreactivity after NaOCl treatment^7^. EDTA is a chelating agent and is therefore responsible for removing the smear layer^10^. Moreover, EDTA was reported to have an antibacterial effect^47,48^, probably due to the chemical chelation involving the external bacterial membrane^48,49^.

The use of NaOCl and EDTA has been recommended during chemo-mechanical reparation^22^. Research has suggested that when in contact with these materials, the dentin may change its physical, chemical and structural properties. These changes may result in decreased dentin microhardness^50^, change its flexural strength^51^ and modulus of elasticity^25,51^, cause irreversible damage of the dentin microstructure^20,52^, and oxidize the organic matrix denaturing the collagen components of the dentin surface^53^. Relating these facts to the possibility of clinical occurrences, degradation of the collagen matrix in mineralized tissues results in a less resistant and more brittle substrate, which can make the endodontically treated teeth more susceptible to crown or root fracture^54^.

In the surface texture analysis presented here, the control group showed less roughness compared to the experimental groups, yet, it showed some similarity to the NaOCl group. This result coincides with surface appearance by the SEM; smear layer is present in both control and NaOCl groups. Indeed, the removal of smear layer at the E, EN and NE groups resulted in the exposure of dentin tubules seen by the SEM images, and was represented by increased roughness of surfaces as measured by the texture surface analysis.

Both EDTA and NaOCl are necessary for the preparation of the root canal; yet the sequence of the irrigation remains debated. It was suggested that when NaOCl is used prior to EDTA, the hydroxyapatite coating appears to shield the collagen fibers from the dissolving effect of NaOCl. However, when NaOCl is used after EDTA, the collagen that was already exposed to demineralizing agents is more intensively subjected to the dissolving effect of NaOCl^49^. In the present study, the sequence of irrigation, did not bear a significant impact on the dentin surface roughness. Yet, a certain tendency was revealed, as higher roughness was detected when NaOCl was used as the final irrigation.

Qian and colleagues^20^ have suggested that when NaOCl is used before EDTA, the average area of tubule openings is smaller since the sequence of root canal wall dentin exposure to the two materials impacts the level of dentin erosion. However, this analysis was conducted on 2D SEM images, and analyzed the difference in the measured area of dentinal openings^20^, whereas in the present study, we addressed the roughness of the surface area in 3D. In our study, although some differences between irrigation protocols could be noted (Table 1), the differences in most of the roughness parameters were not statistically significant.

A recent study by Wang and colleagues^55^ quantified the erosion of the root canal dentin by measuring the atomic percentage in root canal walls via energy-dispersive X-ray spectroscopy. The authors showed significant depletion of calcium and phosphate into the deeper layers of the dentin following a final rinse with NaOCl, while other irrigation protocols caused insignificant changes in atomic composition, suggesting that NaOCl used as a final irrigation solution causes most of the erosion effect. However, in our present study examining superficial surface roughness, most of the measured parameters did not show a significant increase in the surface roughness levels (Table 1).

In order to attain optimal conditions for root canal therapy, the irrigation protocol should result in a disinfected root canal, free of all organic debris, microorganisms and smear layer, while preserving dentin biomechanical properties^22,52,54,56^. Currently, there is insufficient data to conclude whether the change in surface roughness is harmful to the root dentin and the tooth and to determine the resulting effect on the outcome of the endodontic treatment. As is commonly known, the mineral component in hard connective tissues contributes to strength and toughness of the tooth, whereas collagen is responsible for the elastic modulus^49,54,57^. Therefore, any change in dentin structure or configuration (e.g., roughness, thickness of the root, the amount of sclerotic dentin) might serve as a contributing factor in the formation of vertical root fracture^17–19^.

However, for clinical purposes, the softening effect of chemical solutions on root canal surface roughness might be advantageous, as it permits improved preparation and negotiation of tight root canals^58^. Moreover, dentin tubules become more patent and the surface roughness increases, thereby allowing improved micromechanical bonding of endodontic sealers and other materials that require surface irregularities for the adhesive to penetrate and adhere^1,3,5^.

Further research is needed in order to reveal how dentin hardness and its biomechanical properties are affected by different irrigation protocols.

## Conclusions

In the present study, we introduced a new methodology to analyze 3D surface area roughness of dentin after applying different irrigation solutions. The results indicated that although irrigating the root canal using NaOCl and EDTA affects the dentin surface texture by increasing its roughness, the exact sequence of irrigation using these materials has no significant effect. The results indicate that irrigation sequence probably does not affect the mechanical retention. Further study is required in order to determine the effect of irrigation sequence on the chemical retention.

## Data Availability

All data are available from the corresponding author upon reasonable request.
